# Intravenous Nicotinamide Riboside Administration Has a Cardioprotective Effect in Chronic Doxorubicin-Induced Cardiomyopathy

**DOI:** 10.3390/ijms232113096

**Published:** 2022-10-28

**Authors:** Ekaterina Podyacheva, Natalia Semenova, Vsevolod Zinserling, Daria Mukhametdinova, Irina Goncharova, Irina Zelinskaya, Eric Sviridov, Michael Martynov, Svetlana Osipova, Yana Toropova

**Affiliations:** 1 Almazov National Medical Research Centre, Ministry of Health of the Russian Federation, 197341 Saint-Petersburg, Russia; 2 Institute of Biomedical Systems and Biotechnologies, Peter the Great St. Petersburg Polytechnic University, 194021 Saint-Petersburg, Russia

**Keywords:** anthracyclines, doxorubicin-induced cardiomyopathy, fibrosis, intravenous administration, NAD+, nicotinamide riboside, sirtuins

## Abstract

Doxorubicin, which is widely used to treat a broad spectrum of malignancies, has pronounced dose-dependent side effects leading to chronic heart failure development. Nicotinamide riboside (NR) is one of the promising candidates for leveling the cardiotoxic effect. In the present work, we performed a comparative study of the cardioprotective and therapeutic actions of various intravenous NR administration modes in chronic doxorubicin-induced cardiomyopathy in Wistar rats. The study used 60 mature male SPF Wistar rats. The animals were randomized into four groups (a control group and three experimental groups) which determined the doxorubicin (intraperitoneally) and NR (intravenous) doses as well as the specific modes of NR administration (combined, preventive). We demonstrated the protective effect of NR on the cardiovascular system both with combined and preventive intravenous drug administration, which was reflected in a fibrous tissue formation decrease, reduced fractional-shortening decrease, and better antioxidant system performance. At the same time, it is important to note that the preventive administration of NR had a more significant protective effect on the animal organism as a whole. This was confirmed by better physical activity parameters and vascular bed conditions. Thus, the data obtained during the study can be used for further investigation into chronic doxorubicin-induced cardiomyopathy prevention and treatment approaches.

## 1. Introduction

Over the past two decades, oncology treatment approaches have been improved, which has led to an increase in patient survival. However, modern chemotherapeutic drug use is often accompanied by the various severe complication developments associated with nonspecific cytostatic effects [1,2].

Anthracyclines, such as doxorubicin, daunorubicin, epirubicin, and idarubicin, are the basis of many modern polychemotherapy regimens. Currently, they are widely used to treat a broad spectrum of malignancies, including breast cancer, leukemia, lymphomas, sarcomas, and others [3,4]. The drugs of this series are characterized by pronounced dose-dependent cardiotoxic effects [4], leading to left-ventricular remote diastolic dysfunction development and, ultimately, chronic heart failure, which significantly worsens the prognosis and the patient’s quality of life.

There are various mechanisms for the development of cardiotoxic effects in patients receiving anthracycline antibiotics. Oxidative stress involvement [5,6,7] is evidenced by reactive-oxygen-species-induced, or ROS-induced, damage such as lipid peroxidation, DNA destruction, disruption of heart-specific gene expression programs [8], and reduced levels of antioxidants and sulfhydryl groups [9]. Moreover, myofibrillar destruction; apoptosis/necrosis; intracellular-calcium/iron metabolism dysregulation [10,11]; and the disruption of the mitochondria [12,13], sarcoplasmic reticulum [12], and topoisomerase IIβ [14,15] are also important mechanisms in anthracycline cardiotoxic action.

To date, there is no single method for the prevention and treatment of the cardiovascular complications that develop during doxorubicin treatment. The only drug approved by the Food and Drug Administration (FDA) is dexrazoxane [3] which is based on oxidative stress reduction. Scientists are also investigating the cardioprotective effects of angiotensin-converting enzyme, β-blocker carvedilol, and antioxidant resveratrol [3].

Nicotinamide riboside (NR, nicotinamide adenine dinucleotide (NAD+) precursor) is one of the promising candidates for cardiotoxic effect leveling in doxorubicin treatment [16,17]. The importance of NAD+ as a coenzyme consists of its participation in many cellular processes, such as red-ox, catabolic, and anabolic reactions [18], and is also reflected in its cellular balance during the work of the enzymes that actively consume NAD+ (SIRT1-7, PARPs, CD38/157, and SARM1) [19,20]. In this way, it is significant to maintain NAD+(H) levels in a constant balance between its synthesis and consumption in various cellular compartments, such as the nucleus, mitochondria, and cytoplasm.

The information that NR can dose-dependently increase NAD+ levels up to 2.7-fold in mammalian cells with a single oral dose of 1000 mg has led to many studies aimed at the prevention and treatment of cardiovascular complications of various origins [21,22,23]. Taking into account the fact that patients undergoing chemotherapy may acquire many side effects that affect the functioning of various organ systems, including the digestive tract (intestinal mucositis) [24,25], taking the required NR dose orally may lead to insufficient effectiveness due to possible intestinal malabsorption. Chemotherapy-induced intestinal mucositis occurs in 40–100% of all cancer patients receiving chemotherapy drugs [26]. At the same time, developing complications may require a reduction in chemotherapy doses or even the termination of the treatment. It has been shown that after an intravenous doxorubicin infusion, its intestinal concentration is approximately 100 times higher than its concentration in blood plasma [27,28]. Taking into account the pathogenesis of chemo-induced mucositis, NR may be one of the options for a pathogenetically substantiated reduction in doxorubicin-induced intestinal damage [20,29]. In this regard, for the first time in our laboratory, the intravenous NR administration method was used [30], which demonstrated a positive effect on the intestinal wall during ischemia-reperfusion injury development. Therefore, NR intravenous administration could be a good alternative to maintenance therapy for the patients since this route of administration can increase the drug bioavailability and its effectiveness.

The combined use of NR and doxorubicin can lead to chemotherapy toxic-effect decrease due to a significant increase in NAD+ levels, which activates sirtuins. They, in turn, can induce antioxidant defense systems that restore the mitochondrial biogenesis damaged by oxidative stress and promote autophagy activation which is impaired under doxorubicin’s influence [31]. Endothelial SIRT1 activation can control endothelial homeostasis and vascular functioning by modulating the activity of endothelial nitric oxide synthase (eNOS), p53, angiotensin II receptor (ATR2), forkhead box protein O1 (FOXO1), and other mechanisms [32]. Thus, launched mechanisms reduce cardiomyopathy’s severity. Moreover, the supposed synergism of NR and doxorubicin antitumor effects is an interesting hypothesis. SIRT1 activation can prevent tumor vascularization through Notch pathway inhibition and can suppress tumor cell survival by inducing apoptosis in cancer cells through FOXO activation [33,34,35,36]. However, the ways of NR/SIRT1/Notch and NR/SIRT1/FOXO molecular interactions during tumor development still need to be studied in more detail.

In the present work, we performed a comparative study of various intravenous NR administration modes and their cardioprotective effect in chronic doxorubicin-induced cardiomyopathy development in Wistar rats.

## 2. Results

### 2.1. Body Weight and Physical Endurance Assessment

**Figure 1:**
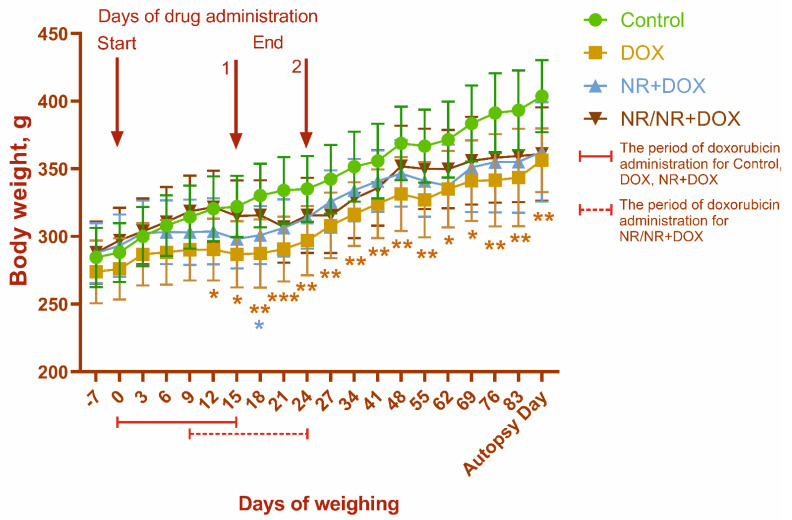
Body−weight dynamics. 1—drug administration endpoint for control, DOX, and NR+DOX groups; 2—drug administration endpoint for NR/NR+DOX group. The first weighing was carried out before the start of each manipulation. During the drug administration, the animals were weighed every 2 days on the day of the substance administration (9 times). At the end of the administration in NR/NR+DOX group, the weight and clinical condition of the animals were monitored once a week. */*—*p* < 0.05, **—*p* < 0.01, and ***—*p* < 0.001 which shows significant difference compared to control group (*—DOX; *—NR+DOX). Data are presented as median [25%; 75%]. Control represents control group (n = 15); DOX represents doxorubicin group (n = 15); NR+DOX represents nicotinamide riboside + doxorubicin group—*combined mode* (n = 15); and NR/NR+DOX represents nicotinamide riboside/nicotinamide riboside + doxorubicin group—*preventive mode* (n = 15).

Death in the animals was not observed during the study. The body-weight dynamics for all the experimental groups are shown in Figure 1. A gradual increase in body weight was observed throughout the experiment for all animals. At the same time, there was a tendency to reduce the body-weight gain in rats of the DOX, NR+DOX, and NR/NR+DOX groups due to drug administration, which indicates the toxic effect of doxorubicin.

**Figure 2:**
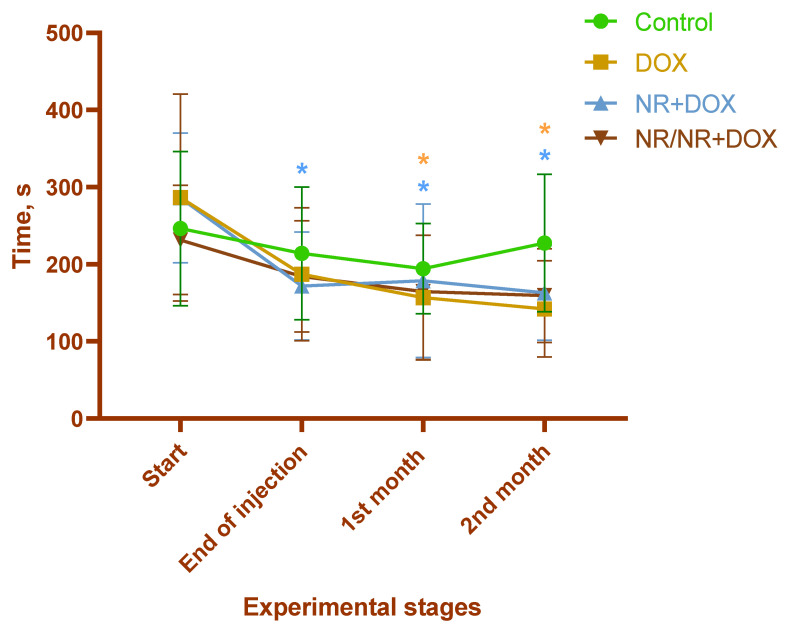
Treadmill activity duration. */*—*p* < 0.05 compared to the initial value (start of the experiment) (*—DOX; *—NR+DOX). Data are presented as median [25%; 75%]. Control represents control group (n = 15); DOX represents doxorubicin group (n = 15); NR+DOX represents nicotinamide riboside + doxorubicin group—*combined mode* (n = 15); and NR/NR+DOX represents nicotinamide riboside/nicotinamide riboside + doxorubicin group—*preventive mode* (n = 15).

The exercise tolerance significantly decreased within 1 month after the final drug administration in the rats of the DOX and NR+DOX groups (*p* < 0.05 compared to the initial value (Start)) (Figure 2). No changes were observed in the control and NR/NR+DOX groups. Table 1 shows the change in physical activity by group as a percentage of the baseline (100%) at the start of the experiment.

### 2.2. Echocardiography

**Figure 3:**
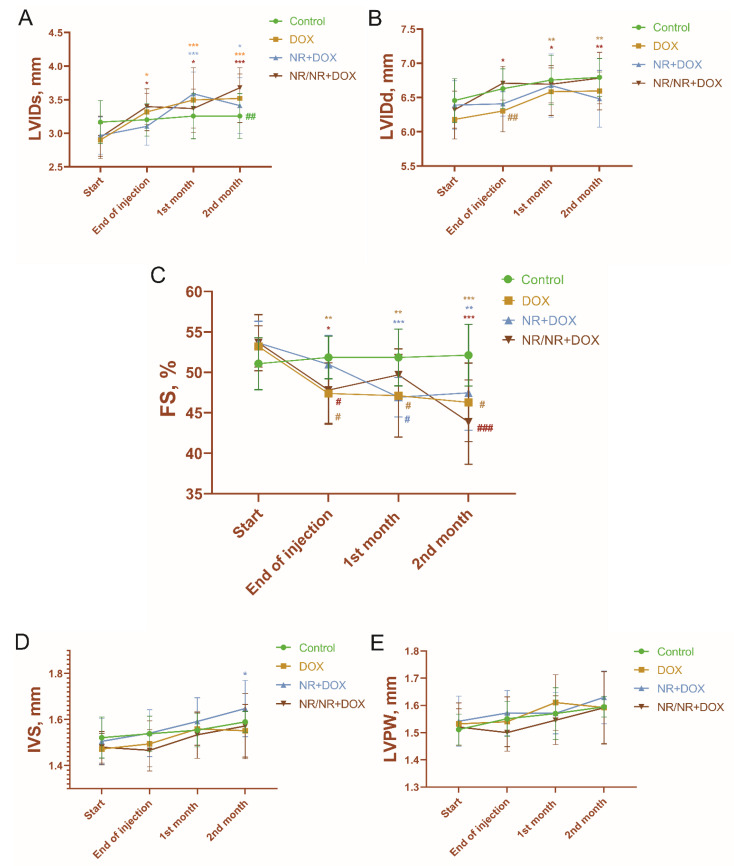
Left-ventricular parameters according to ECHO. (**A**)—left-ventricular end-systolic internal diameter, or LVIDs; (**B**)—left-ventricular end-diastolic internal diameter, or LVIDd; (**C**)—fractional shortening, or FS; (**D**)—anterior wall thickness, or IVS; and (**E**)—posterior wall thickness, or LVPW. */*/*—significant difference compared to the start of the experiment (*—DOX; *—NR+DOX; and *—NR/NR+DOX); #/#/#—significant difference in FS between DOX, NR+DOX, and NR/NR+DOX groups compared to control group; and #/#/#—significant difference in LVIDs and LVIDd between control, DOX, and NR+DOX groups compared to NR/NR+DOX group (#—DOX; #—NR+DOX; and #—NR/NR+DOX). (*/*/*—*p* < 0.05, **/**/**—*p* < 0.01, and ***/***/***—*p* < 0.001; #/#/#—*p* < 0.05, ##/##—*p* < 0.01, and ###—*p* < 0.001.) Data are presented as median [25%; 75%]. Control represents control group (n = 15); DOX represents doxorubicin group (n = 15); NR+DOX represents nicotinamide riboside + doxorubicin group—*combined mode* (n = 15); NR/NR+DOX represents nicotinamide riboside/nicotinamide riboside + doxorubicin group—*preventive mode* (n = 15).

There were significant increases in the left-ventricular end-systolic internal diameter (LVIDs) and end-diastolic internal diameter (LVIDd) of the DOX and NR/NR+DOX groups (Figure 3A,B). At the same time, the animals of the NR+DOX group had an increase only in LVIDs (*p* < 0.05, compared to baseline, compared to control). These changes led all the experimental groups to a significant fractional-shortening (FS) decrease (Figure 3C). The most pronounced decrease in FS (19% of the initial value) was observed in the rats of the NR/NR+DOX group. In contrast, the animals of DOX group had a 14% decrease, and those of the NR+DOX had a 12% decrease. These changes reflected impaired-cardiac-muscle diastolic function as a subclinical cardiotoxicity manifestation.

**Figure 4:**
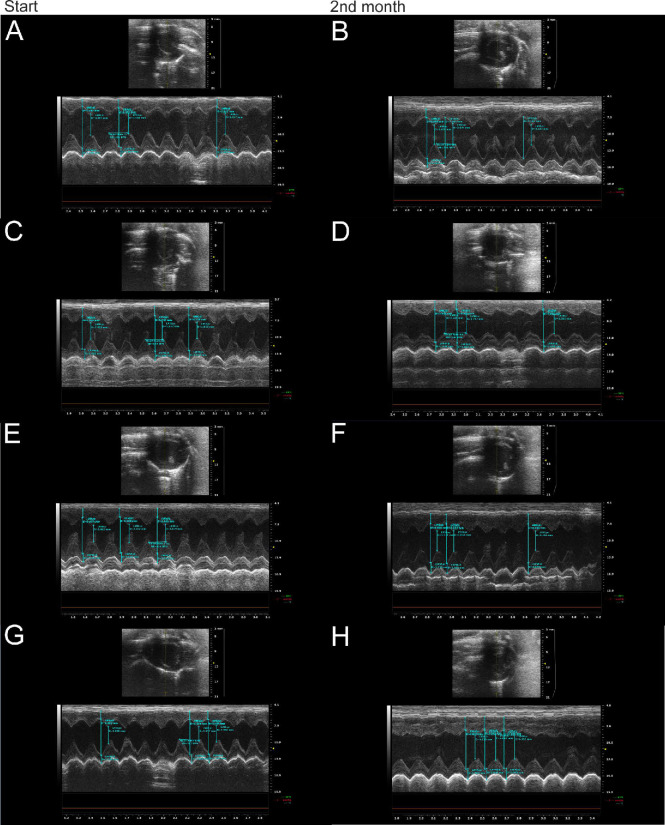
Representative ECHO photographs of control, DOX, NR+DOX, and NR/NR+DOX groups at the start of the experiment and two months after doxorubicin administration completion. (**A**,**B**)—control group; (**C**,**D**)—DOX group; (**E**,**F**)—NR+DOX group; and (**G**,**H**)—NR/NR+DOX group. DOX represents doxorubicin group; NR+DOX represents nicotinamide riboside + doxorubicin group—*combined mode*; and NR/NR+DOX represents nicotinamide riboside/nicotinamide riboside + doxorubicin group—*preventive mode*.

The left-ventricle anterior wall thickness (IVS) was increased in the NR+DOX group (*p* < 0.05, compared to the initial value, compared to control (Figure 3D)). The posterior wall thickness (LVPW) had no significant changes in all the groups of the animals (Figure 3E). The representative ECHO photographs of the four groups at the start of the experiment and two months after drug administration completion are shown in Figure 4.

### 2.3. Clinical Blood Tests

There were no significant changes observed in the blood parameters between the control, DOX, and NR+DOX groups throughout all the timepoints of the assessment. However, at the end of the drug administration, several parameters (LYM, RBC, HGB, HCT, PLT, and WBC) were significantly reduced, and PLT increased in NR/NR+DOX compared to both the control and DOX groups (Table S1), which was restored to normal levels with time. While the clinical significance of blood parameter alterations uniquely in this preventive approach is unknown, it is duly noted that NR generally has low toxicity even when administered orally at high doses [22,37]. Further studies should investigate the differential effects of NR administration modalities.

### 2.4. Chemiluminescent Analysis of ROS

Two months after drug administration ending, a decrease in the time to reach the luminescence intensity maximum (Tmax) was observed in the animals of the DOX group only (Table 2). Simultaneously, during zymosan-induced chemiluminescence, which characterizes the rate of “respiratory burst” development and reflects the specific phagocyte response to a stimulus (zymosan), Tmax was significantly higher in the rats of the NR+DOX and NR/NR+DOX groups compared to the control group. In contrast, the maximum luminescence intensity value (Imax) was significantly higher in DOX group samples. The activation index (AI) showed hyperactivation in doxorubicin administration only compared to the control group. The hyperactivation of antioxidant-reserve enzymatic blood systems in the animals treated with NR was not observed (Table 2).

### 2.5. Morphological Analysis

**Figure 5:**
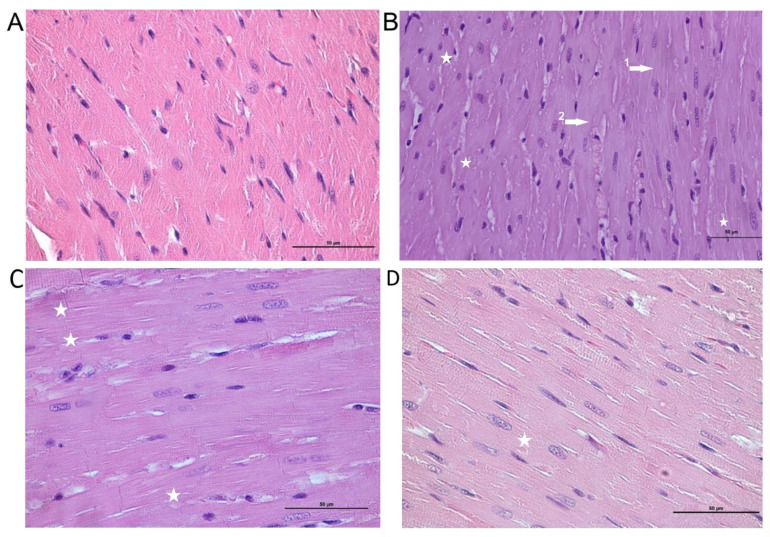
Cardiac muscle tissue. (**A**)—control group, normal structure. (**B**)—DOX group, dystrophic changes in cardiomyocytes: loss of striation (1,2 arrows) and vacuolization of the cytoplasm (stars). (**C**)—NR+DOX group, minimal alterative changes. (**D**)—NR/NR+DOX group, minimal alterative changes. Stained with hematoxylin–eosin, ×50. DOX represents doxorubicin group; NR+DOX represents nicotinamide riboside + doxorubicin group—*combined mode*; and NR/NR+DOX represents nicotinamide riboside/nicotinamide riboside + doxorubicin group—*preventive mode*.

Mild dystrophic changes in cardiomyocytes with cytoplasm vacuolization and loss of striation were noted in the myocardial tissue histo-architectonics in the DOX group (Figure 5). Additionally, a capillary plethora of varying severity was revealed, but there was no inflammatory infiltration. In part of the observations, there were cells with signs of apoptosis; however, large necrosis foci were not visualized. Mild dystrophic changes were also noted in the NR+DOX and NR/NR+DOX groups (Figure 5).

**Figure 6:**
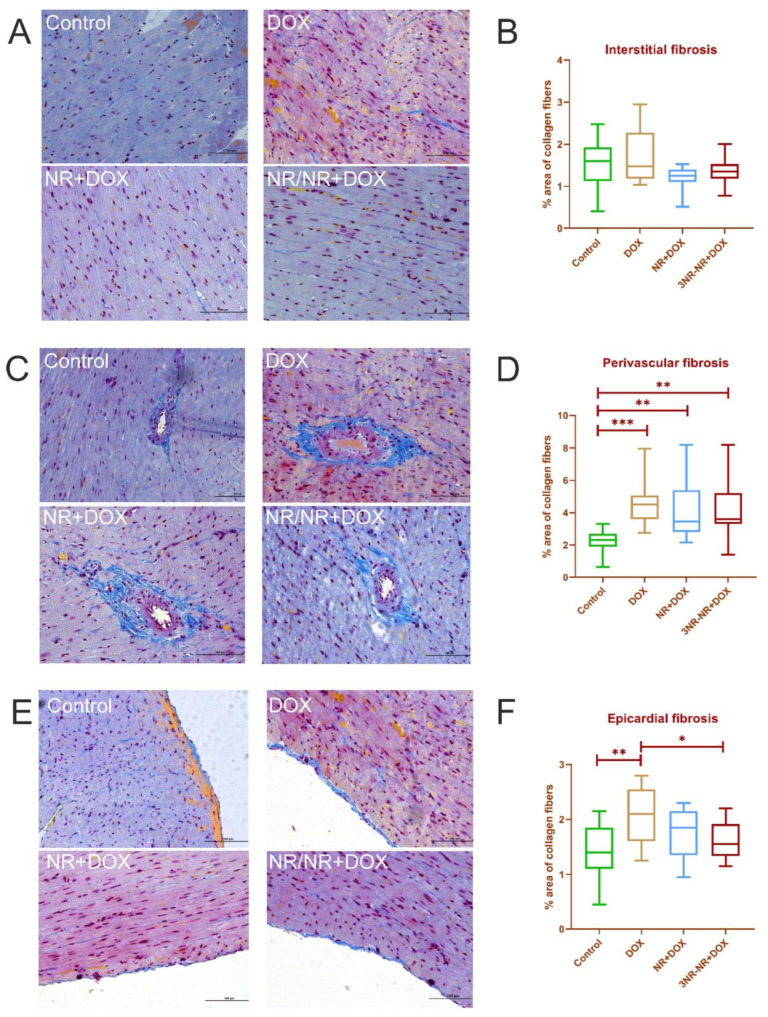
Morphometric values (% area of collagen fibers) in cardiac muscle. (**A**,**B**)—interstitial fibrosis; (**C**,**D**)—perivascular fibrosis; (**E**,**F**)—epicardial fibrosis. Collagen fibers are blue. Staining by Mallory, × 100. *—*p* < 0.05, **—*p* < 0.01, and ***—*p* < 0.001. Data are presented as median [25%; 75%]. Control represents control group (n = 15); DOX represents doxorubicin group (n = 15); NR+DOX represents nicotinamide riboside + doxorubicin group—*combined mode* (n = 15); and NR/NR+DOX represents nicotinamide riboside/nicotinamide riboside + doxorubicin group—*preventive mode* (n = 15).

The results for Mallory’s histochemical stain morphometry are shown in Figure 6. There were no significant differences in the groups in the myocardium. However, a significant increase in the collagen percentage of the samples of the experimental groups compared to the control was revealed near the vessels. NR made these changes less pronounced. There was a significant increase in fibrosis in the DOX group compared to the animals of the control and NR/NR+DOX groups in the pericardial zone. A significant increase in the total collagen area was observed in all the experimental groups compared to the control. The most pronounced increase was noted in the animals of the DOX group (Figure 6). NR made the total collagen score decrease; this decrease had a significant difference in the NR+DOX group compared to the DOX group.

### 2.6. Mesenteric Artery Functional Activity

**Figure 7:**
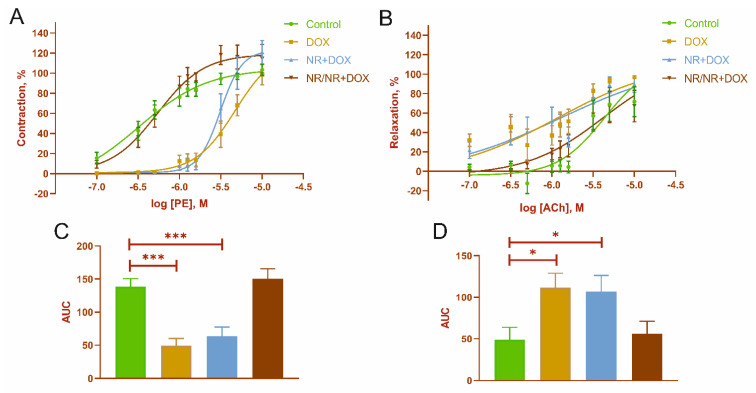
Mesenteric artery functional activity. (**A**)—dose-dependent response curves to phenylephrine (PE); (**B**)—dose−dependent response curves to acetylcholine (ACh); (**C**)—area under the curves (AUC) for PE-dependent response; and (**D**)—AUC for ACh-dependent response. *—*p* < 0.05, and ***—*p* < 0.001, significant difference compared to control group. Values represent mean ± SEM mean from 9 arteries of 3 rats in every group (n = 36). Control represents control group; DOX represents doxorubicin group; NR+DOX represents nicotinamide riboside + doxorubicin group—*combined mode*; and NR/NR+DOX represents nicotinamide riboside/nicotinamide riboside + doxorubicin group—*preventive mode*.

Dose-dependent response curves are shown in Figure 7. The curve parameters are presented in Table 3. There was a decrease in the vascular response to phenylephrine (PE) and an increase in response to acetylcholine (ACh) in the animals of the DOX and NR+DOX groups (Figure 7A,B). The maximum contractile and relaxation responses in all the experimental groups did not differ when using the concentrations of PE and ACh (10 μM) (Table 3). Also, the DOX and NR+DOX groups have a lower sensitivity to PE. A decrease in the AUC was noted in the DOX and NR+DOX groups compared to the control for PE (Figure 7C). At the same time, these groups showed increased integral responses to ACh (Figure 7D).

## 3. Discussion

In this study, we intended to compare different nicotinamide riboside intravenous administration modes regarding its cardioprotective effect in chronic doxorubicin-induced cardiomyopathy development. For this study, we chose two injection modes—combined and preventive. They were chosen based on the doxorubicin dose-dependent damaging effect. That is, the combined administration mode was based on the simultaneous use of NR and the chemotherapeutic drug. The preventive administration mode suggested early NR accumulation in cells before chemotherapy treatment followed by simultaneous NR and doxorubicin administration. One of the most important tasks was the complex physical and physiological assessment of doxorubicin toxic action. For these purposes, we used such instrumental investigations as a clinical blood test, chemiluminescent analysis, activity time on treadmill, echocardiography, and weighting. These tests can be performed in living animals and provide us with plenty of data for the physical state and activity endurance, cardiac function, hematopoiesis, and ROS production. However, there is no option to avoid post-mortem investigation, so the histological assessment of cardiac muscle allowed us to look at the fibrotic changes underlying functional disfunction. Additionally, the wire myography of mesenteric arteries reflected the ability of resistance arteries in adequate blood supply regulation. A two-month-long observation period after drug treatment let us analyze the changes at different recovery stages and compare the effects of preventive and combined NR administration on the recovery process.

Both NR administration modes did not prevented reduced weight gaining. Simultaneously, the NR/NR+DOX group started to lose weight later than the NR+DOX group. Treadmill activity assessment showed a significant decrease in exercise tolerance in the DOX and NR+DOX groups but not the NR/NR+DOX group during the observation time. In a recent study, it was shown that NR, by increasing NAD+ in muscle tissue, stimulates the growth of slow fibers, increases the endurance on the treadmill, and prevents the decline in grip strength [38]. Moreover, improvements in basal and maximal cellular aerobic and anaerobic respiration were observed in mouse myoblasts. Preclinical data in 2022 showed that small NR supplementation in the early postnatal period beneficially affects lipid and energy metabolism in adult skeletal muscles and the liver through activation of sirtuin 1 and AMP-dependent protein kinase signaling [39]. Therefore, it can be assumed that NR administration before chemotherapy has a positive effect on an animal’s physical condition.

Simultaneously, the NR/NR+DOX group of the animals was characterized by more pronounced temporal changes in the general blood test parameters compared to the NR+DOX group. Generally, the clinical condition of both groups of animals was similar. The blood–antioxidant system assessment showed a significant decrease in the integral spontaneous response in the NR/NR+DOX group but not in the NR+DOX group. At the same time, both NR-treated groups showed a higher time to achieve the maximum-induced response. The presented spontaneous and induced antioxidant system changes in NR-treated groups were not enough to evoke changes in AI. Therefore, both NR administration modes are efficient for antioxidant system maintenance. It is known that oxidative stress, caused by an increasing ROS-formation level during doxorubicin chemotherapy, is one of the main mechanisms of cardiotoxicity. NR is able to reduce free radical formation and reduce oxidative damage in the myocardium [40,41]. Our chemiluminescence analysis results confirmed this.

ECHO showed significant changes in the cardiac function of the experimental animals. LVIDs increased in both NR groups, but, in NR+DOX, this change came late and had less of a resulting amplitude. The NR/NR+DOX group had an LVIDd that was close to the control. In the results, all DOX-treated groups had an FS decrease. At the same time, there was no significant difference in FS between the NR+DOX group and the control group. It was also interesting that only the NR+DOX group had an increase in anterior wall thickness. The study of Zheng et al. also showed an FS decrease and the necrosis present in the hearts of mice treated with NR together with doxorubicin [40]. However, these damages were less pronounced and were determined by the administrated NR dose. The dose of 500 mg/kg was the most efficient in mice. Therefore, it can be assumed that 6-times the administration of 300 mg/kg has an insufficiently pronounced effect on the myocardium with the same doxorubicin administration regularity. It is necessary to provide additional research aimed at the most effective NR dose selection.

Cardiac fibrosis was more pronounced in the pericardium and around the vessels (mainly the arteries) than between cardiomyocytes due to many stromal cells in these areas that produce collagen. In total, NR administration has some ability to decrease fibrosis severity. Many studies have shown that oral NR administration breaks off cardiac dysfunction development in a mouse model of dilated cardiomyopathy [42], prevents metabolic disorder development, and reduces the left-ventricular myocardium fibrosis level in a model of myocardial infarction in mice [43,44]. These improvements are explained by the fact that NR addition stimulates an increase in Nmrk expression and sirtuin activity restoration (for example, SIRT1 and SIRT3), which is necessary for normal mitochondrial biogenesis, and, at the same time, it contributes to PARP1 expression decrease. In our study, a significant decrease in myocardium contractile function was observed in the animals treated with NR, but simultaneously, a decrease in the collagen percentage was revealed. These effects of the various intravenous administration modes of the NAD+ precursor need to be studied in more detail at the molecular level, which is the goal of our further research.

Changes in mesenteric artery functional activity were especially interesting. NR had a vasoprotective effect only in the preventive administration mode (the NR/NR+DOX group), and, in this case, contraction and relaxation activities were almost identical to those in the control group. However, in the combined administration mode (the NR+DOX group), NR gave no vasoprotective effect. Presumably, this fact indicates preactivated protective vascular mechanisms (for example, activation of endothelial SIRT1-dependent signaling pathways), which, in the end, could help cope with the toxic effect of doxorubicin. Moreover, it can be assumed that the preliminary NR administration contributed to the early rearrangement of NAD+ synthesis from nicotinamide phosphoribosyltransferase (NAMPT) to nicotinamide riboside kinase (NRK). This change helped to more quickly compensate for the energy depletion of cells with doxorubicin introduction. The Nmrk2 gene is also an AMPK-sensitive gene [45]. Therefore, NR administration activates the AMPK pathway, which is aimed at conserving energy in cells and, consequently, improving cellular metabolism in general. In this study, the mesenteric vessels were a reflection of the systemic endothelial response to the drug effects. It is known that doxorubicin has a damaging effect on the endothelium of both myocardial and systemic vessels. The chemotherapy drug decreases tight junction formation through occluded-zone-1 (or ZO-1) expression reduction. It has a negative effect on the nitric oxide (NO) level through enzymatic inhibition, lowering the endothelin-1 (or ET-1) level, and ROS accumulation [32,46]. All these disorders lead to endotheliocyte death in different vascular beds, including cardiac, which stimulates fibroblasts activity, consequently increases vessel wall rigidity, and contributes to perivascular fibrosis formation.

Thus, we demonstrated that NR intravenous administration decreases cardiac-muscle fibrous-tissue formation in chronic doxorubicin-induced cardiotoxicity development. To date, the beneficial NR effect on the liver and white adipose tissue fibrosis reduction has been reported [47,48,49]. Moreover, a pronounced protective effect of NR on the cardiovascular system was demonstrated both with combined and preventive intravenous drug administration. Simultaneously, the preventive NR administration had a more significant protective effect on the animal’s body as a whole. This was confirmed by better physical activity parameters and bloodstream conditions (mesenteric artery functional activity).

Today, NR studying has a great perspective due to its ability to quickly normalize the NAD+/NADH ratio in cardiac muscle via NRK 1/2, and NR also shows protective effects in adverse cardiac remodeling [45,50]. Additionally, the intracellular NAD+ increase increases SIRT activity, which is able to protect from oxidative damage due to antioxidant-defense-system stimulation [51,52,53], mitochondrial biogenesis restoration, and cell cycle arrest, and, respectively, suppresses apoptotic mechanisms through p53 and FOXO regulation [31,35,54]. Therefore, sirtuin stimulation contributes to cardiac hypertrophy reduction, metabolic dysregulation, and cardiac inflammation. However, a more detailed study of NAD+/SIRT regulation mechanisms is needed, particularly in the field of their relationship with other vital cellular enzyme systems that consume NAD+, such as PARPs. Additionally, further study is needed to determine the pharmacokinetics and pharmacodynamics of therapeutic intravenous NR in animal models for effective translation of the cardioprotective effects to humans.

In this work, there are no data comparing the oral and intravenous routes of NR administration aimed at its ability to reduce doxorubicin-induced intestinal damage. NR-only animals were not presented separately in this study. Since the purpose of this work was to confirm the pharmacological NR effect on the myocardium, this limitation did not reduce the quality and validity of the findings. Moreover, it is important to note that treadmill, ECHO, and blood sampling were performed at a different timepoint in the NR/NR+DOX group. However, this discrepancy was due to the differences in the dosing regimen and did not influence the results, as they were conducted in a timely manner after their respective doxorubicin injury.

The data obtained during the study are of applied value and can be used to develop approaches for chronic doxorubicin-induced cardiomyopathy prevention and treatment.

## 4. Materials and Methods

### 4.1. Pharmacological Agents

Doxorubicin-LENS^®^, 50 mg (VEROPHARM, LLC, Belgorod, Russia), and nicotinamide riboside (Kingherbs Limited, China) were prepared for administration immediately before use (ex tempore). Drug dosages were determined empirically, considering existing literature data [9,22,40,41,55] as well as our own unpublished data. Doses of doxorubicin and NR were based on animal weight. The drugs were diluted in 0.9% saline. During the experiment, doses were adjusted considering animals weight changes.

### 4.2. Ethical Approval

Animal study protocol was approved by an institutional animal care and use committee from the Centre for Experimental Biomodeling, Institute of Experimental Medicine, Almazov National Medical Research Centre, Ministry of Health of the Russian Federation (№21-14П3#V1).

### 4.3. Experimental Protocol

**Figure 8:**
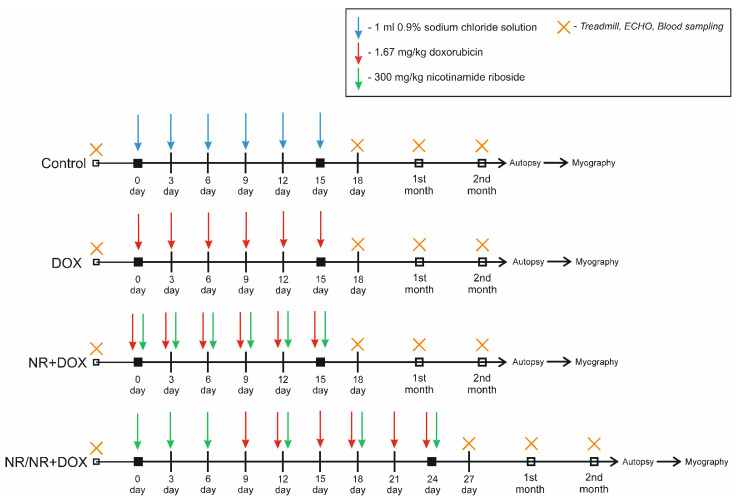
Experimental design.

The study used 60 mature male SPF Wistar rats weighing 283 ± 22 g. Animals were housed in a barrier-type facility in standard environmental conditions: 12 h light/dark cycle, standard temperature and humidity, and were administered food and water ad libitum. The experimental animal choice was made in accordance with existing published data on doxorubicin cardiomyopathy modeling. It was also important to obtain the proper amount of biological samples for comprehensive drug effect assessment [9]. Firstly, animals were randomly placed into 4 experimental groups (Table S2). Control group animals were administered 1 mL of 0.9% sodium chloride solution intraperitoneally 6 times every 2 days. DOX group animals were injected intraperitoneally with 1.67 mg/kg of doxorubicin 6 times every 2 days (10 mg/kg cumulative dose). The combined mode consisted of 6 intravenous injections of 300 mg/kg of NR (1800 mg/kg cumulative dose) 30 min (half-life) before intraperitoneal administration of 1.67 mg/kg of doxorubicin every 2 days. The preventive mode consisted of 3-time intravenous injection of 300 mg/kg of NR every 2 days then 6-time intraperitoneal injection of 1.67 mg/kg of doxorubicin every 2 days with 3-time alternations every other time of the administration of 300 mg/kg of NR (30 min before the doxorubicin administration). The time schedule for drug administration is shown in Figure 8.

Animals were under observation for 2 months after reaching the cumulative dose. This time was necessary for delayed-doxorubicin-effect implementation, such as myocardial fibrosis and diastolic dysfunction. At all experimental stages, treadmill parameters were recorded to assess the physical endurance of animals; echocardiography (ECHO) to assess myocardial contractility; and blood samples, which were also taken from the retroorbital sinus for hematological analysis and chemiluminescence, to assess ROS generation (in the 2nd month). Intravital blood sampling from the retroorbital sinus, in amount of 0.25 mL (before autopsy 0.5 mL), was performed under isoflurane anesthesia. Autopsy was performed on the animal under xylasine-zoletyl anesthesia (10 mg/kg of xylasine + 25 mg/kg of zoletyl, intramuscularly), and the heart was removed for morphological analysis. Before the cardiac arrest, 10% KCl solution was injected into the left ventricle until cardiac arrest was completed in the diastole phase. To study the functional activity of the vessels, the mesentery was taken from three animals of each group for further wire myography.

The animals were weighed every two days from the start until the end of drug administration then were weighed once a week (DX 1200 WP, AND, Tokyo, Japan).

### 4.4. Physical Tolerance

Physical tolerance was examined using a treadmill (Treadmill System for rats, TSE, Germany) at a movement rate of the transporter line of 0.7 m/s and a slope angle of 15°. Full animal tiredness was asserted by lack of reaction to electric shock (2.5 MA). The end of the running time was reported in seconds, as previously described [56].

### 4.5. Echocardiography

Before echocardiography, animal was anesthetized using an inhalation mixture of 1.7% isoflurane—98% oxygen. Echocardiographic images were obtained with a heart rate stabilized at ∼400 beats/minute. Echocardiograms were recorded using the Vevo^®^ 2100 (VisualSonics Inc., Toronto, Ontario, Canada). All parameters were taken from the right parasternal short axis. Myocardium functioning parameter analysis was carried out in the M mode. Left-ventricular end-diastolic internal diameter at Q wave (LVIDd), left-ventricular end-systolic internal diameter at T wave (LVIDs), posterior wall thickness at diastole (LVPW), and anterior wall thickness at diastole (IVS) were measured. Another parameter was calculated as follows: left-ventricular short-axis fractional shortening FS (%) = (LVIDd − LVIDs)/LVIDd × 100.

### 4.6. Morphological Analysis

Tissue samples of heart were fixed in formalin according to the routine procedure. Dehydration and paraffin embedment were carried out in an automatic histological processor Vip5Jr (Sakura, Japan) in the prepared IsoPREP solution (Biovitrum, Russia) and HISTOMIX paraffin medium (Biovitrum, St. Petersburg, Russia). Sections 2–3 μm thick were prepared using a rotary microtome HM 325 (Thermo, Waltham, Massachusetts, USA) and were subsequently deparaffinized, dehydrated, and stained with hematoxylin–eosin according to the manufacturer’s recommendations (Biovitrum, St. Petersburg, Russia).

Microscopic examination and photorecording were carried out on a Nis-E microscope (Nikon, Tokyo, Japan) at ×40, ×100, ×200, and ×400 magnifications.

To determine the degree of collagen fibrosis, histochemical staining was performed according to Mallory (Biovitrum, St. Petersburg, Russia). The histological evaluation algorithm was as follows: Several representative visual fields were photographed at one magnification of 400× (4 fields of view for each area of interest) in each histological preparation. Fibrosis area was assessed using Nis-Elements (Nikon, Tokyo, Japan) and Orbit morphometry software as a percentage of visual field area (which was always equal to 100%) to assess perivascular and interstitial fibrosis.

To standardize the assessment of perivascular fibrosis, only vessels (arteries) of medium caliber, cut in a cross section, and of approximately the same diameter were selected. Before photographing, the vessel was fixed in the most central area of the field of view, including all collagen fibers surrounding the vessel in the image.

Evaluation of epicardial fibrosis was done differently. In the photographs of this area, part of the field of view was represented by cardiac tissue with epicardium, and part of the field of view was empty. When photographing, the field of view was selected in such a way that approximately 50% of the area was occupied by the cardiac tissue of the area of interest. When evaluating these images in the morphometric program, cardiac tissue with epicardium was isolated, and % fibrosis was evaluated in the selected area. The empty part of the image was not included in the assessment. The data were entered into tables—each measurement separately entered, then the average value for each area was calculated separately for each preparation along with the average value for the group.

“Total” fibrosis is the sum of the average values for each area, and it calculated for each preparation separately along with the average value for the group.

### 4.7. Hematology

A general blood test was performed using an Abacus Junior Vet (Diatron, Budapest, Hungary) veterinary hematological analyzer. The following quantitative parameters were evaluated: white blood cells (WBC, g/L); leukocyte fraction—granulocytes (Gr, %); lymphocytes (Lym, 10^9^ g/L); monocytes (Mi, %); red blood cells (RBC, ×10^12^/L); hemoglobin (Hb, g/L); and erythrocyte indices including mean concentration hemoglobin (MCH, pg), mean corpuscular hemoglobin concentration (MCHC, g/L), mean corpuscular volume (MCV, fl), hematocrit (Hct, %), and platelets (PLT, ×10^9^/L).

### 4.8. Chemiluminescent Analysis

Blood was obtained by sampling from the retroorbital sinus under isoflurane anesthesia before the autopsy of the animal in a volume of 250 µL. The production of ROS by blood cells was assessed using the chemiluminescent method. Luminol (2.2 × 10^−4^ M) was used as a chemiluminescence enhancer. Zymosan solution was used to evaluate the reserve capacity for activation of blood enzymatic systems.

Registration of ROS during spontaneous and induced chemiluminescence was carried out using a 12-channel Lum-1200 chemiluminometer (DISoft, Moscow, Russia) for 60 min. Based on the registered data, the following parameters were calculated:

- The maximum value of the glow intensity (Imax, $\frac{pulses}{s}\times 10^{3}$);
- The time to reach the maximum value of the glow intensity (Tmax, min);
- Area under the curve (S, pulses).

Zymosan-induced enhancement of chemiluminescence was assessed using the activation index (AI) which was calculated as the ratio of the area under the induced chemiluminescence curve to the area under the spontaneous chemiluminescence curve.

### 4.9. Blood Vessel Functional Activity Assessment

The vascular function of mesenteric arteries (n = 12) was studied using a wire myograph DMT 620M (Danish Myo Technology, Hinnerup, Midtjylland, Denmark) as described previously [57]. During the necropsy, mesentery was removed and placed in a Petri dish filled with ice-cool Krebs–Henseleit solution of the following composition (mM): 119 NaCl, 4.7 KCl, 1.17 KH_2_PO_4_, 1.6 CaCl_2_, 1.2 MgSO_4_, 25 NaHCO_3_, 5.5 glucose, 0.03 EDTA, and pH 7.4. Further manipulations to isolate the mesenteric arteries were performed using a dissecting microscope (MSP-1, LOMO, Russia). Three third-ordered arteries were isolated from the mesentery.

The blood vessels were mounted in the myograph chamber using two 40 μm wires. After transmural pressure normalization, contractile mechanisms were activated by incubation in high-potassium Krebs–Henseleit solution ((mM): 78.2 NaCl, 60 KCl, 1.17 KH_2_PO_4_, 1.6 CaCl_2_, 1.2 MgSO_4_, 25 NaHCO_3_, 5.5 glucose, and 0.03 EDTA) and 10 μM phenylephrine (PE), followed by repeated washing with Krebs–Henseleit solution. To study vasocontraction, cumulative dose-dependent response protocol to phenylephrine was used. The vessels were incubated in solution with a phenylephrine concentration from 10^−7^ to 10^−5^ M. To study endothelium-dependent relaxation, the vessels were preliminarily contracted with phenylephrine by 60% of the maximum. Then, incubation with acetylcholine (ACh) was performed according to a scheme similar to the contractile response. The data were recorded using LabChart 8 software (ADInstruments Ltd., Oxford, UK). For dose-dependent curves, concentration providing 50% of the maximum response (EC50, μM), maximal response (Emax, %), and area under the curve (AUC) were calculated.

### 4.10. Statistical Analysis

All calculations were performed with GraphPad Prism 8 (GraphPad Software, Inc., San Diego, CA, USA) and LibreOffice (The Document Foundation, Berlin, Germany) software for Windows (Microsoft Inc., Redmond, Washington, DC, USA). The Shapiro–Wilk test was used to detect the normal distribution. An unpaired, non-parametric Kruskal–Wallis test with Dunn’s test was used to compare several independent groups since the data obtained (ECHO, hematological values, chemiluminescence values, and the histological differences between different groups) were non-normally distributed. The Wilcoxon test was used for pairwise comparison of dependent values of the non-normal distribution (treadmill data, ECHO data, *—dynamics compared to the initial value). Nonlinear regression was used for myography data. The values in the groups were processed using nonparametric statistics (median, 25th, and 75th percentiles (Me (25%; 75%))). Differences were considered significant at *p* < 0.05.

## Figures and Tables

**Table 1:** Changes in the physical activity of animals as a percentage of the initial value.

	Start	End of Injection	1st Month	2nd Month
Control	100%	87%	79%	94%
DOX	100%	65% *	55% *	50% *
NR+DOX	100%	61%*	64% *	58% *
NR/NR+DOX	100%	80%	71%	69%

DOX represents doxorubicin; NR represents nicotinamide riboside. *—*p* < 0.05, significant difference compared to the initial value (start of the experiment). Control represents control group (n = 15); DOX represents doxorubicin group (n = 15); NR+DOX represents nicotinamide riboside + doxorubicin group—*combined mode* (n = 15); and NR/NR+DOX represents nicotinamide riboside/nicotinamide riboside + doxorubicin group—*preventive mode* (n = 15).

**Table 2:** Results of spontaneous and induced chemiluminescence.

Parameter	Control	DOX	NR+DOX	NR/NR+DOX
**Spontaneous chemiluminescence**				
**Tmax, min**	37.8 [17.6; 51.0]	17.6 [15.2; 39.3] **	40.6 [16.2; 52.6]	37.8 (26.6; 43.8)
**Imax,** $\frac{\mathrm{pulses}}{s}\times 10^{3}$	0.031 [0.027; 0.033]	0.028 [0.027; 0.033]	0.029 [0.027; 0.033]	0.029 [0.027; 0.031]
**S_1_, pulses**	1.78 [1.64; 1.94]	1.89 [1.46; 2.22] *	1.81 [1.64; 1.96]	1.69 [1.64; 1.81] *
**Zymosan-induced chemiluminescence**				
**Tmax, min**	11.4 [7.3; 25.8]	15.6 [7.3; 27.9]	29.0 [9.3; 34.6] **	20.1 [12.2; 36.2] *
**Imax,** $\frac{\mathrm{pulses}}{s}\times 10^{3}$	0.113 [0.067; 0.165]	0.156 [0.081;0.353] *	0.096 [0.052; 0.206]	0.083 [0.044; 0.179]
**S_2_, pulses**	6.63 [4.73; 9.88]	13.59 [5.73; 21.14] **	7.05 [3.30; 15.28]	6.37 [3.21; 12.76]
**AI**	3.47 [2.67; 6.09]	8.07 [2.38; 13.87] **	4.09 [1.82; 9.04]	3.87 [1.78; 7.915]

AI represents the activation index; Imax represents glow intensity maximum; Tmax represents time to reach glow intensity maximum; and S represents area under the curve. *—significant difference compared to control group (*—*p* < 0.05, **—*p* < 0.01). Data are presented as median [25%; 75%]. Control represents control group (n = 15); DOX represents doxorubicin group (n = 15); NR+DOX represents nicotinamide riboside + doxorubicin group—*combined mode* (n = 15); and NR/NR+DOX represents nicotinamide riboside/nicotinamide riboside + doxorubicin group—*preventive mode* (n = 15).

**Table 3:** Dose-dependent curve parameters.

Agonist	Parameter	Control	DOX	NR+DOX	NR/NR+DOX
**PE**	logEC_50_, M	−6.48 ± 0.31	−5.32 ± 0.15 **	−5.5 ± 0.04 **	−6.26 ± 0.12
	Emax, %	103.29 ± 5.48	98.588 ± 10.07	120.86 ± 11.69	118.17 ± 14.11
**ACh**	logEC_50_, M	−5.6 ± 0.08	−5.67 ± 0.11	−5.896 ± 0.10	−5.73 ± 0.12
	Emax, %	71.69 ± 15.46	97.00 ± 1.04	89.31 ± 8.18	67.21 ± 16.26

**—*p* < 0.01, significant difference compared to control group. Values represent mean ± SEM mean from 9 arteries of 3 rats in every group (n = 36). Control represents control group; DOX represents doxorubicin group; NR+DOX represents nicotinamide riboside + doxorubicin group—*combined mode*; and NR/NR+DOX represents nicotinamide riboside/nicotinamide riboside + doxorubicin group—*preventive mode*.

## Data Availability

Not applicable.

## Supplementary Materials

The supporting information can be downloaded at https://www.mdpi.com/article/10.3390/ijms232113096/s1.
