# Supplementary material for: Intravenous Nicotinamide Riboside Administration Has a Cardioprotective Effect in Chronic Doxorubicin-Induced Cardiomyopathy
Source: Int J Mol Sci. 2022 Oct 28;23(21):13096. doi: 10.3390/ijms232113096 (PMC9653852; doi:10.3390/ijms232113096)
Supplement: Supplementary file 1 [file ijms-23-13096-s001.zip › Table S1.pdf]

Table S1. Quantitative parameters of hematological analysis.

Time	Groups			
	Control	DOX	NR+DOX	NR/NR+DOX
		**WBC,10^9 g/l**		
**Start**	8,4 [8,2; 8,9]	8,3 [7,8; 8,9]	10,2 [8,9; 11,8]	8,7 [5,1; 14,8]
**End of injection**	9,3 [6,9; 11,9]	10,4 [8,5; 12,3]#	9,1 [6,8; 10,8]	6,6 [4,9; 6,9]
**1st month**	10.1 [8.7; 11.3]	11.4 [10.1; 12.1]#	10.6 [8.7; 11.4]	8.9 [7.1; 10.1]
**2nd month**	9.4 [8.5; 11.1]	8.7 [7.1; 11.2]	9.7 [8.1; 10.7]	9.2 [6.6; 10.8]
		**LYM, 10^9 g/l**		
**Start**	6,5 [4,9; 7,7]	4,8 [4,8; 5,6]	5,9 [5,1; 7,7]	5,5 [4,1; 10,9]
**End of injection**	7,6 [4,7; 8,1]	6,5 [5,6; 7,7] #	5,7 [4,5; 7,1]	3,8 [3,3; 4,8]*
**1st month**	6.4 [5.7; 7.1]	7.8 [6.4; 8.6]	7.2 [5.8; 8.1]	5.9 [5.4; 6.35]
**2nd month**	6.8 [5.1; 7.6]	5.7 [4.3; 6.9]	6.1 [4.5; 7.7]	5.1 [4.1; 6.7]
		**GRA, 10^9 g/l**		
**Start**	2,2 [1,7; 3,2]	2,4 [2,1; 3,5]	2,8 [2,4; 3,3]	2,5 [1,1; 3,2]
**End of injection**	2,8 [1,1; 3,8]	2,8 [1,9; 4,3]	2,6 [2,1; 3,3]	1,8 [0,9; 2,5]
**1st month**	2.2 [1.6; 3.5]	2.9 [2.6; 3.4]	2.7 [2.2; 3.3]	2.1 [1.4; 3.1]
**2nd month**	2.4 [1.7; 3.7]	2.3 [1.9; 4.3]	2.6 [1.7; 4.2]	2.5 [1.7; 3.1]
		**MI%**		
**Start**	5,9 [2,1; 10,9]	12,8 [3,5; 13,8]	11,8 [9,5; 13]	10,2 [5,1; 11,5]
**End of injection**	2.4[1.0;6.7]	2.4[0.7; 6.1]	1.9[0.6; 6.3]	2.9 [0.7; 10.6]
**1st month**	7.1 [4.3; 10.2]	5.0 [3.6; 9.3]	4.2 [0.8; 8.1]	6.3 [2.2; 11.5]
**2nd month**	6.1 [4.9; 7.6]	4.2 [1.3; 6.5]	6.5 [4.2; 8.2]	5.8 [2.9; 6.9]
		**RBC, 10^12/l**		
**Start**	7,8 [7,4; 8,4]	8,1 [7,8; 8,3]	8,1 [7,8; 8,4]	7,9 [7,3; 8,2]
**End of injection**	8.8 [7.6; 9.1]	7.8 [7.6; 8.7]	7.7 [7.4;7.9]	7.1 [6.6;7.2]*
**1st month**	8.6 [8.1;8.8]	8.1 [7.7;8.5]	8.2 [7.6; 8.4]	8.3 [8.0; 8.6]
**2nd month**	7.6 [7.3; 8.2]	8.1 [7.1; 8.7]	8.1 [7.8; 8.4]	8.2 [7.8; 8.6]
		**HGB, g/l**		
**Start**	165,5 [161,3; 170,5]	171,5 [165,8; 175]	170,5 [167,5; 173,3]	173 [157; 174]
**End of injection**	173.0 [152.0;184.0]	167.0 [158.5; 181.5]	162.5 [157.5;164.0]	135.5 [132.0; 140.8]*
**1st month**	163.0 [158.0; 172.0]	170.0 [159.5; 182.5]	167.0 [162.5; 177.3]	169.0 [161.0; 175.0]
**2nd month**	160.5 [149.5; 171.0]	167.0 [150.5; 173.5]	167.0 [154.5; 171.5]	173.5 [167.8; 176.8]
		**HCT%**		
**Start**	38,9 [36,2; 39,8]	38,9 [38,1; 39,4]	38,9 [37,9; 40,4]	36,4 [35,5; 37,8]
**End of injection**	39.6 [33.7; 40.7]	39.4 [36.4;40.9]#	37.3 [35.6; 38.6]	31.2 [30.6;33.4]*
**1st month**	38.6 [36.5;39.2]	38.5 [37.7; 40.9]	40.4 [37.9; 42.8]	38.6 [38.1; 39.4]
**2nd month**	35.3 [33.1; 36.9]	37.6 [34.3; 40.4]	37.7 [35.3; 39.1]	37.86 [36.3; 38.5]
		**MCV, fl**		
**Start**	49,5 [46,5; 50]	48 [47,25; 49,5]	49 [47,75; 49,25]	46 [46; 49]
**End of injection**	44.0 [42.5; 47.0]	48.0 [46.2; 50.0]##	47.5 [46.2; 48.7]	45.5 [44.7; 46.0]

**1st month**	45.0 [44.0; 47.0]	47.0 [47.0; 50.5]	50.0 [49.3; 51.3]	46.0 [45.5; 48.0]
**2nd month**	45.0 [44.0; 45.0]	47.00 [45.00; 49.50]	46.0 [45.5; 48.0]	46.0 [45.0; 47.0]
**MCH, pg**				
**Start**	21,2 [19,9; 21,8]	21 [20,7; 21,8]	21,15 [20,9; 21,7]	21,7 [21,1; 21,9]
**End of injection**	19.5 [19.2;20.4]	20.9 [20.2; 21.5]##	20.6 [20.1; 21.1]	19.4 [19.1; 20.0]
**1st month**	19.7 [19.4;20.1]	20.8 [20.4;21.7]	20.3 [19.8; 21.6]	20.4 [19.6; 20.9]
**2nd month**	20.7 [20.3; 21.2]	20.7 [19.9; 21.0]	20.5 [20.1; 21.1]	21.3 [20.8; 21.7]
**MCHC, g/l**				
**Start**	427 [418,8; 441,5]	441 [435,5; 444,3]	436,5 [427,8; 449,3]	457 [443; 478]
**End of injection**	446.0 [432.0;452.5]	432.5 [424.0; 441.0]	433.0 [420.0; 446.5]	432.0 [422.0; 439.5]
**1st month**	439.0 [427.0;448.0]	432.0 [414.0; 447.0]	415.0 [395.3; 428.5]	436.0 [417.5; 444.0]
**2nd month**	460.5 [450.3; 470.8]	438.0 [427.5;446.0]	443.0 [427.5;458.5]	463.0 [454.5; 474.5]
**PLT, 10^9 g/l**				
**Start**	607[431,5; 639]	591,5[579; 604,8]	543 [396; 600,5]	580,5 [468,6; 614,7]
**End of injection**	628.0 [529.0;771.0]	689.5 [469.3; 806.8]#	788.0 [672.5;1011]	1044 [1011; 1235]*
**1st month**	558.0 [506.5; 607.5]	594.5 [548.5; 680.5]	742.0 [489.5; 842.5]	621.0 [473.0; 736.0]
**2nd month**	533.0 [514.3; 595.8]	666.0 [642.5; 819.0]	639.5 [453.0; 850.0]	725.5 [662.3; 796.0]**

GR, granulocytes; HB, hemoglobin; HCT, hematocrit; LYM, lymphocytes; MCH, mean concentration hemoglobin; MCHC, mean corpuscular hemoglobin concentration; MCV, mean corpuscular volume; MI, monocytes; PLT, platelets; RBC, red blood cells; WBC, white blood cells. * - compared to Control group; # - compared to NR/NR+DOX (* - $p<0.05$, ** - $p<0.01$). Control, control group; DOX, doxorubicin group; NR+DOX, nicotinamide riboside+doxorubicin group - *Combined mode*; NR/NR+DOX, nicotinamide riboside/nicotinamide riboside+doxorubicin group - *Preventive mode*. Data are presented as Median [25%;75%].
