# Supplementary material for: Intravenous Nicotinamide Riboside Administration Has a Cardioprotective Effect in Chronic Doxorubicin-Induced Cardiomyopathy
Source: Int J Mol Sci. 2022 Oct 28;23(21):13096. doi: 10.3390/ijms232113096 (PMC9653852; doi:10.3390/ijms232113096)
Supplement: Supplementary file 1 [file ijms-23-13096-s001.zip › Table S2.pdf]

Table S2. Characteristics of the experimental groups.

Group, n	Drugs	Administration route	Dose	Administration regimen	Cumulative dose	Duration
**Control (n = 15)**	0.9% sodium chloride solution	i.p.	1 ml	6 times every two days	6 ml	2.5 weeks
**DOX (n = 15)**	doxorubicin	i.p.	1,67 mg/kg	6 times every two days	10 mg/kg	2.5 weeks
**NR+DOX (n = 15)**	NR + doxorubicin	i.v. + i.p.	300 mg/kg + 1,67 mg/kg	*Combined mode*<br>6 times every two days: i.v. NR → 30 min → i.p. doxorubicin	1800 mg/kg + 10 mg/kg	2.5 weeks
**NR/NR+DOX (n = 15)**	NR + doxorubicin	i.v. + i.p.	300 mg/kg + 1,67 mg/kg	*Preventive mode*<br>3 times every two days i.v. NR → i.p. doxorubicin 6 times alternating every other time with NR (3 times)	1800 mg/kg + 10 mg/kg	3.5 weeks

DOX, doxorubicin; i.p., intraperitoneal administration; i.v., intravenous administration; NR, nicotinamide riboside. 10mg/kg of doxorubicin in rats is equivalent to 1.6mg/kg in humans (HED). 1800 mg/kg NR in rats is equivalent to 290.3 mg/kg in humans (HED).
